# Antimicrobial, Anticancer and Multidrug-Resistant Reversing Activity of Novel Oxygen-, Sulfur- and Selenoflavones and Bioisosteric Analogues

**DOI:** 10.3390/ph13120453

**Published:** 2020-12-11

**Authors:** Małgorzata Anna Marć, Annamária Kincses, Bálint Rácz, Muhammad Jawad Nasim, Muhammad Sarfraz, Carlos Lázaro-Milla, Enrique Domínguez-Álvarez, Claus Jacob, Gabriella Spengler, Pedro Almendros

**Affiliations:** 1 Department of Medical Microbiology and Immunobiology, University of Szeged, H-6720 Szeged, Hungary; kincses.annamaria90@gmail.com (A.K.); balintracz95@gmail.com (B.R.); spengler.gabriella@med.u-szeged.hu (G.S.); 2 Division of Bioorganic Chemistry, School of Pharmacy, Saarland University, D-66123 Saarbruecken, Germany; jawad.nasim@uni-saarland.de (M.J.N.); s8musarf@stud.uni-saarland.de (M.S.); c.jacob@mx.uni-saarland.de (C.J.); 3 Grupo de Lactamas y Heterociclos Bioactivos, Unidad Asociada al CSIC, Departamento de Química Orgánica I, Facultad de Ciencias Químicas, Universidad Complutense de Madrid, E-28040 Madrid, Spain; carloslazaromilla@ucm.es; 4 Instituto de Química Orgánica General IQOG-CSIC, Consejo Superior de Investigaciones Científicas, Juan de la Cierva 3, 28006 Madrid, Spain; e.dominguez.alvarez@csic.es (E.D.-Á.); palmendros@iqog.csic.es (P.A.)

**Keywords:** selenium, flavonoids, sulfur, multidrug resistance (MDR), MDR efflux pumps, P-glycoprotein (P-gp) efflux pump, nematicidal activity, antimicrobial, cancer

## Abstract

Multidrug resistance of cancer cells to cytotoxic drugs still remains a major obstacle to the success of chemotherapy in cancer treatment. The development of new drug candidates which may serve as P-glycoprotein (P-gp) efflux pump inhibitors is a promising strategy. Selenium analogues of natural products, such as flavonoids, offer an interesting motif from the perspective of drug design. Herein, we report the biological evaluation of novel hybrid compounds, bearing both the flavone core (compounds **1**–**3**) or a bioisosteric analogue core (compounds **4**–**6**) and the triflyl functional group against Gram-positive and Gram-negative bacteria, yeasts, nematodes, and human colonic adenocarcinoma cells. Results show that these flavones and analogues of flavones inhibited the activity of multidrug resistance (MDR) efflux pump ABCB1 (P-glycoprotein, P-gp). Moreover, the results of the rhodamine 123 accumulation assay demonstrated a dose-dependent inhibition of the abovementioned efflux pump. Three compounds (**4**, **5**, and **6**) exhibited potent inhibitory activity, much stronger than the positive control, verapamil. Thus, these chalcogen bioisosteric analogues of flavones become an interesting class of compounds which could be considered as P-gp efflux pump inhibitors in the therapy of MDR cancer. Moreover, all the compounds served as promising adjuvants in the cancer treatment, since they exhibited the P-gp efflux pump modulating activity.

## 1. Introduction

The emergence of multidrug resistance (MDR) towards cytotoxic drugs is still a major problem in cancer therapy. One of the most widespread mechanisms of resistance involves the efflux of drug molecules out of the cells. P-glycoprotein (P-gp or ABCB1) discovered in 1970 is a member of the highly conserved integral membrane-bound protein transporters of the ATP-binding cassette (ABC) transporter family. These ABC transporters are present in the tissues of the gastrointestinal tract, liver, and lungs; they are comprised of two ATP-binding sites and 12 transmembrane domains which constitute a drug-binding pocket [1,2]. P-gp mainly recognizes amphipathic cationic compounds as substrates [3]. These proteins are localized in several barriers, such as the blood–brain barrier (BBB), blood-cerebrospinal fluid (B-CSF), blood–retina barrier (BRB), blood–testis barrier (BTB), and in the placenta [1,2]. Moreover, P-gp is implicated in the development of MDR by playing an important role in some drug–drug interactions [3]. This protein serves as an ATP-dependent efflux pump, removing both endogenous molecules and xenobiotics from the cells and decreasing their intracellular concentrations. It is a natural protective protein of the cells, which protects them against the toxic action of xenobiotic compounds [3,4]. In humans, this transporter is encoded by the *MDR1* (multidrug resistance 1) gene [3]. Intriguingly, one of the most frequent mechanisms underlying MDR appearance is the overexpression of MDR transporters, which enables the active extrusion of a broad range of anticancer drugs out of the cancer cells. In this context, P-gp serves as a resistance mediator, which can recognize and expel a broad spectrum of chemotherapeutic agents, e.g., daunorubicin, vinblastine, vincristine, epirubicin, etoposide, imatinib, irinotecan, paclitaxel, or colchicine [5,6]. Allen et al. reported that P-gp, together with the multidrug resistance-associated protein 1 (MRP1), is the major determinant of the innate drug sensitivity even at the lowest level of expression [7,8]. Thus, the design of inhibitors of the efflux pumps, especially of the P-gp, is a promising strategy in cancer therapy [3,6,9].

Flavonoids are an important class of natural products which contain a polyphenolic core in their chemical structure and are commonly present in plants. Flavonoids comprise different classes of compounds, such as flavones, isoflavones, flavonols, chalcones, and anthocyanins. Amongst flavonoids, the flavones are the most important subgroup and are frequently found in vegetables, fruits, grains, bark, roots, stems, flowers, tea, coffee, beer, and wine [10,11]. The flavone scaffold is the key structural motif involved in redox modulation and other biological activities, especially in the treatment of oxidative stress-related diseases [12,13,14]. Moreover, these heterocycles are well known for their beneficial effects on human health and have been widely exploited in the pharmaceutical industry [12,13,14,15]. These naturally occurring heterocycles exhibit a wide range of biological activities, such as anti-tumor, anti-microbial, antioxidant, anti-inflammatory, anti-diabetic, anti-hypertensive, anti-atherogenic, gastro-protective, anti-platelets, or anti-thrombotic and estrogenic activities [12,13,14,15,16]. 

Moreover, the flavone motif is attracting much attention in the development of novel agents in medicinal chemistry, especially for the treatment of multi-factorial diseases [15]. There is a renewed interest in the preparation of the flavone-based organic compounds [12,13,14], and the resulting compounds present promising biological activities. A taxol-linked flavone, for instance, was reported to improve the anti-tumoral character of the resulting compound [17]. The anticancer activities of flavonoid derivatives are based on different mechanisms, such as the disruption of the cellular migration, the induction of cell cycle arrest and apoptosis, the modulation of the nuclear receptor responsiveness, and the inhibition of the angiogenesis [10]. Besides, flavones are potent known inhibitors of the serine/threonine protein kinases and the tankyrase, which are potential drug targets of cancer treatment [10,17]. Moreover, some of these flavonoids were selected for clinical trials as potential protein kinase inhibitors [10]. 

The inclusion of fluorinated scaffolds is a profitable approach to enhance both the chemical and the biological properties of the resulting organic molecules. Recently, a considerable interest has been developed in heterocyclic compounds bearing the trifluoromethylsulfonyl (triflyl) moiety (Tf−SO_2_CF_3_). Triflyl is one of the strongest electron-withdrawing existing functional groups which confers a mild lipophilicity to the molecules. Consequently, the preparation of hybrid compounds bearing both the flavone core and the triflyl group is rather interesting [18].

**Figure 1:**
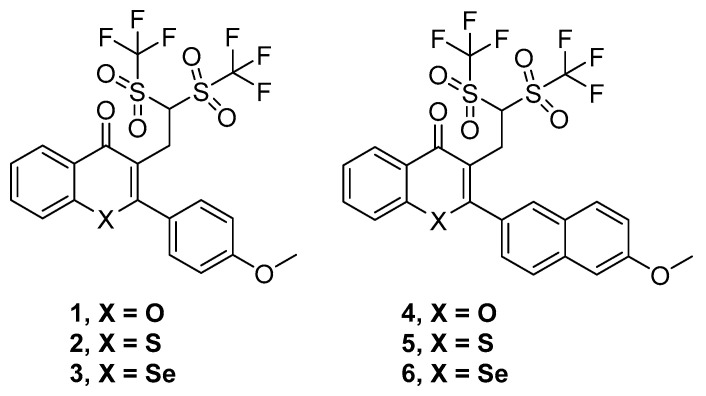
Structures of the flavone derivatives (**1**–**3**) and the bioisosteric analogues of flavones (**4**–**6**).

Thus, six compounds that were synthesized and previously described [18] have been tested herein in different cancer, bacterial, and MDR biological assays. These compounds were the ditriflyl-substituted flavone **1** and its naphthyl bioisosteric derivative **4**. Besides, the sulfur and selenium isosteres of **1** and **4** were also considered. These three flavones and three bioisosteric analogues of flavones are shown in Figure 1. For the sake of brevity, we will denote them in this text as flavones or flavone derivatives, keeping in mind that compounds **4**–**6** are bioisosteric analogues of flavones. 

## 2. Results

### 2.1. Cytotoxic and Antiproliferative Activity

The results of the MTT assays with human Colo 205 and Colo 320 cell lines revealed the inactivity of all the tested flavones, thioflavones, and selenoflavones since the IC_50_ values of all tested flavones ranged above 100 µM (Table 1). In addition, the compounds were found to be non-toxic to MRC-5 normal lung cell lines. Unfortunately, none of the flavone compounds presented IC_50_ values comparable to the ones of doxorubicin (Table 1). 

Similarly, the compounds did not exhibit any significant antiproliferative activity, since the IC_50_ values were too high (i.e., more than 100 µM) when the compounds were tested against colonic adenocarcinoma cell lines (Table 2). 

### 2.2. Antibacterial and Antifungal Activities

The antimicrobial activity was evaluated against a broad spectrum of microorganisms including bacteria (Gram-positive bacterium *S. carnosus* and Gram-negative bacterium *E. coli*) and pathogenic and non-pathogenic yeasts (*C. albicans* and *S. cerevisiae*, respectively). 

A concentration-dependent increase in the antibacterial activity against both Gram-positive and Gram-negative bacteria was observed for compounds **2**–**5**. The highest activity against *S. carnosus* was observed for compound **3**, which inhibited the growth by 57% followed by compounds **2** and **5**, which inhibited the growth by 47%, and compound **4**, which reduced the growth of *S. carnosus* by 37% at the highest concentration of 200 µM. Compound **3** showed the highest antimicrobial activity against *E. coli* and inhibited the growth by 49%. Similarly, compounds **4** and **2** resulted in a decrease in the growth by 45% and 42%, respectively. Compounds **5** and **1** inhibited the growth of *E. coli* by 39% and 30%, respectively. None of the compounds exhibited antimicrobial activity against the yeast cells, even at higher concentrations (Figures S1–S6). 

### 2.3. Nematicidal Activity

**Figure 2:**
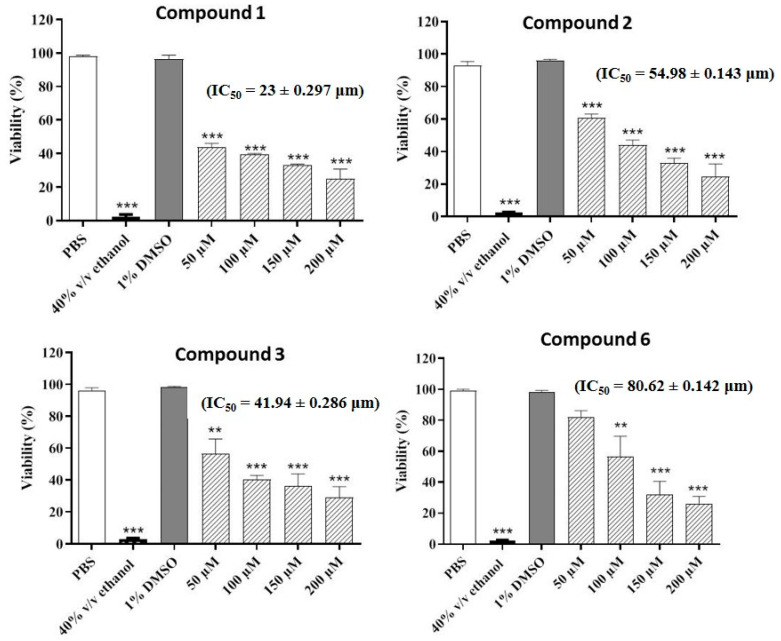
Concentration-dependent increase in nematicidal activities of the most active compounds. Phosphate buffered saline (PBS) and ethanol (70% *v/v*) were employed as negative and positive controls, respectively. Values represent mean ± S.D. *** *p* < 0.001 and ** *p* < 0.01.

The compounds were also evaluated for their potential nematicidal activities. The results of the nematicidal assay revealed that four compounds (**1**–**3** and **6**) decreased the viability of the nematodes in a concentration-dependent manner. The highest nematicidal activity was observed for compound **2**, which decreased the viability to 24%, followed by compound **6**, which reduced the viability to 26% at the highest concentration. Compounds **1** and **3** decreased the viability to 30% and 29%, respectively at the highest concentration (200 µM) (Figure 2). 

### 2.4. MDR Efflux Pump Inhibition

**Figure 3:**
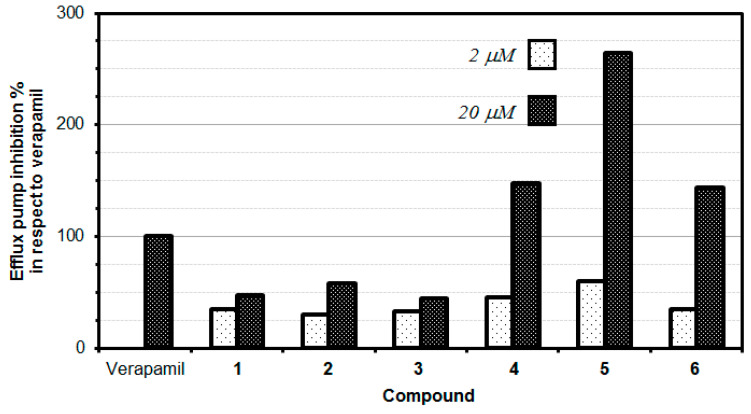
Efflux pump inhibition of novel flavonoids and bioisosteric analogues.

The P-glycoprotein (P-gp) inhibitory properties of the tested flavone derivatives were evaluated in a rhodamine R123 accumulation assay in two different concentrations. This fluorescence assay is commonly employed to identify the inhibitors of P-gp efflux pump activity. R123 belongs to specific fluorescent substrates of P-gp. The impact of tested compounds on P-gp efflux pump activity was examined by comparing the retained fluorescence of samples treated with compounds to the P-gp activity in the presence of reference compound-verapamil, which is a known P-gp inhibitor (100% efflux pump inhibition). The results revealed a dose-dependent inhibition of P-gp MDR efflux pump activity manifested by the tested flavone derivatives. Compounds with FAR values greater than 1 were considered to be active P-gp inhibitors, whilst compounds with FAR values greater than 10 were considered to be strong MDR modulators [19]. Intriguingly, the higher concentration of each tested compound resulted in an increased inhibition against multidrug resistance. Three of the tested flavone derivatives (bis(triflyl)flavone (**4)**, bis(triflyl)thioflavone (**5**) and bis(triflyl) selenoflavone (**6)**) exerted an excellent inhibitory effect, which was stronger than verapamil (Table 3, Figure 3). 

## 3. Discussion

None of the compounds displayed antiproliferative or cytotoxic activity in any of the two cancer cell lines and the non-cancer cell line evaluated at concentrations below 100 μM. These compounds are still very interesting from the perspective of their activities against the resistant cells, since they strongly inhibited the P-gp MDR efflux pump. This effect is achieved at concentrations at which these flavone derivatives are non-toxic. Thus, this fact could open a novel approach in the fight against MDR resistance.

The data showed that the thioflavone derivative **5** turned out to be the most active at 20 µM concentration, with an MDR efflux pump inhibiting activity close to up to 264%, which means that compound **5** inhibited the P-gp 2.64-fold stronger than the reference (verapamil). Moreover, compounds **4** and **6** also significantly inhibited the P-gp protein by 147.50% and 143.33%, respectively, at the concentration of 20 µM, still higher than the activity observed for verapamil. Interestingly, at a lower concentration of 2 µM, none of the tested compounds provided an inhibitory activity comparable to the reference drug (verapamil). It is, however, interesting to note that the thioflavone derivative (**5**) demonstrated the highest inhibitory effect (59.92%) at the concentration of 2 µM, this inhibition being comparable to the one exerted by the reference drug verapamil (Table 3, Figure 3). 

For the two series of compounds evaluated (phenyl-substituted chalcogen flavones **1**, **2**, and **3**) and naphthyl-substituted chalcogen flavones (**4**, **5**, and **6**), it was observed that the respective thioflavone derivatives exhibited a higher efflux pump inhibition than its corresponding oxygen and selenium isosteres. Although the difference in the P-gp efflux pump inhibitory effect of oxygen and selenium derivatives is minute, oxygen derivatives are slightly more active than the selenium ones. Intriguingly, the increase in the aromaticity obtained when switching from the phenyl-substituted chalcogen flavones to the naphthyl-substituted chalcogen flavones, a significant increase in the inhibitory activity of the efflux pump, i.e., 3.14-fold, 4.57-fold, and 3.23-fold was observed for naphthyl flavone, naphthyl thioflavone, and naphthyl selenoflavone, respectively, when compared with the corresponding phenyl flavone, phenyl thioflavone, or phenyl selenoflavone derivative. This fact indicates that the modifications in the chemical structure can significantly alter the inhibition activity and suggests that appropriate modifications could improve the activity to lead to more potent and effective P-gp inhibitors. This should be studied in the next works, as could lead to novel, potent and selective efflux pump inhibitors. 

Since the flavonoid compounds were not toxic towards cancer (Colo 205 and Colo 320) and non-tumour (MRC-5) cell lines at the concentration of 20 μM in the rhodamine assay, it can be assumed that the most active flavone compounds could reveal their promising inhibitory activity at a non-toxic concentration. Furthermore, the fact that the compounds did not show cytotoxicity at concentrations below 100 μM provides the opportunity of exploiting such agents in future therapies as promising non-toxic co-adjuvants of chemotherapy drugs. Future studies could explore whether the combined administration of these compounds (and/or their appropriately modified derivatives) and clinical chemotherapy drugs are able to enhance the anticancer activity of the chemotherapy drugs. Taking into account the potential antioxidant nature of flavonoids, designing tailored novel dual antioxidant-coadjuvant molecules is an interesting approach from the perspective of drug design. 

Taken together, all of the tested flavonoids can be successfully proposed as P-gp efflux pump inhibitors in the future therapy of MDR colon cancer. The most potent P-gp inhibitors include the naphthyl thioflavone **5**, the naphthyl flavone **4**, and the naphthyl selenoflavone **6**. In addition, such compounds could be exploited as a safe non-toxic adjuvant of chemotherapy drugs since none of the compounds presented cytotoxicity at concentrations below 100 μM. 

Regarding the antimicrobial activity, none of the compounds presented antibacterial or antifungal activity. Nevertheless, they exhibited a significant nematicidal activity particularly at high concentrations (≤ 200 μM). This activity is not potent for clinical uses (an activity at concentrations below 10 μM would be considered effective). The current investigation, however, could be a good starting point to improve the activity of the tested compounds through the design of more effective nematicidal flavone-containing compounds. Additionally, these compounds may have an application in other fields such as the veterinary field or crop protection in the agriculture arena. 

## 4. Materials and Methods 

### 4.1. Tested Flavone Derivatives and Bioisosteric Analogues

The synthesis of compounds **1**–**6** was previously reported [18]. All of these compounds were obtained with good purity (purity > 95% as confirmed by LC/MS) for biological screening. Compounds were initially dissolved in DMSO to obtain their respective stock solutions. 

The final working solutions were achieved by diluting the corresponding stock solution in the appropriate volumes of culture medium. Concentration of DMSO was adjusted to ensure that all the working solutions employed had a DMSO concentration below 1%.

### 4.2. Reagents

Analytical grade (to enable its use without further purification) rhodamine 123 (R123); sodium dodecyl sulfate (SDS), 3-(4,5-dimethylthiazol-2-yl)-2,5-diphenyltetrazolium bromide (MTT) and dimethyl sulfoxide (DMSO) were acquired at Sigma-Aldrich (St. Louis, MO, USA), whereas verapamil was acquired at EGIS Pharmaceutical Company (Budapest, Hungary). 

The stock solution of rhodamine was prepared in phosphate buffered saline whereas verapamil was simply dissolved in water. The solutions were freshly prepared before each assay.

### 4.3. Cell Lines

Three cell lines have been used in this work: (i), the doxorubicin-sensitive Colo 205 (ATCC-CCL-222) human colonic adenocarcinoma cell line; (ii) the multidrug resistant Colo 320/MDR-LRP expressing P-gp (MDR1)-LRP (ATCC-CCL-220.1) human colonic adenocarcinoma cell line; and the MRC-5 human embryonal lung fibroblast cell line (ATCC CCL-171). The three cell lines were purchased from LGC Promochem, Teddington, UK. 

Their culture conditions are the following ones: 

Colo 205 (ATCC-CCL-222) and Colo 320/MDR-LRP expressing P-gp (MDR1)-LRP (ATCC-CCL-220.1 human colon adenocarcinoma cell lines. As previously described [20], the cells were cultured in RPMI 1640 medium supplemented with 10% heat-inactivated fetal bovine serum, 2 mM *L*-glutamine, 1 mM Na-pyruvate, and 100 mM Hepes. The cell lines were incubated at 37 °C, in a 5% CO_2_, 95% air atmosphere. The semi-adherent human colon cancer cells were detached with Trypsin-Versene (EDTA) solution for 5 min at 37 °C [20].

MRC-5 human embryonal lung fibroblast cell line (ATCC CCL-171). As previously described [20], the cells were cultured in Eagle’s Minimal Essential Medium (EMEM, containing 4.5 g/L of glucose) supplemented with a non-essential amino acid mixture, a selection of vitamins and 10% heat-inactivated fetal bovine serum. The cell lines were incubated at 37 °C, in a 5% CO_2_, 95% air atmosphere [20].

### 4.4. Cytotoxic and Antiproliferative Activity

The influence of synthetic flavonoid derivatives (compounds **1**–**6**) on the doxorubicin-sensitive Colo 205, multidrug resistant Colo 320/MDR-LRP adenocarcinoma and MRC-5 cell lines was evaluated employing the MTT metabolic activity assay, using a procedure described in Supplementary Materials and in previous works [20], and applying the appropriate formulas to calculate the cell growth inhibition [21] and the selectivity index (SI) [22], as well as the criterion to determine whether a compound is strongly or moderately selective [22].

Briefly, the serial dilutions in culture medium required for the MTT metabolic assay (ranging from 100 to 0.195 µM) were freshly prepared before the assay. The stock solutions (10 mM) of the tested compounds were prepared in pure DMSO and the final concentration of DMSO was adjusted to less than 1% threshold in the biological experiments. The concentration-dependent growth inhibition was evaluated in 96-well flat-bottomed microtiter plates. Doxorubicin (DOXO) was employed as a positive control whilst culture medium containing cells was used as a negative control [20]. 

Prior to the experiments, the adherent human embryonal lung fibroblasts (MRC-5) were cultured in flat-bottomed microtiter 96-well plates in an EMEM media supplemented with 10% heat-inactivated fetal bovine serum. Cells were seeded at a density of 10.000 cells per well for 24 h before the beginning of the experiment. The cells were cultured at 37 °C in the humidified cell incubator with the 5% of CO_2_ and 95% air environment. The medium was subsequently removed from the plates containing the cells, and the dilutions of tested compounds, previously made in a separate micro-titration plate, were added to the cells (at final volume equal to 200 μL) [20].

In the case of the colonic adenocarcinoma cells, two-fold serial dilutions of the flavone compounds were prepared in 100 μL of RPMI 1640, horizontally. Furthermore, the semi-adherent colonic adenocarcinoma cells were treated with Trypsin-Versene (EDTA) solution. Then, cells were adjusted to a density of 5 × 10^3^/well for antiproliferative assay and 1 × 10^4^/well for the estimation of cytotoxic activity in 100 µL of medium. The cells were added to each well except to the medium control wells. The culture plates were subsequently incubated at 37 °C for 24 h and 72 h, respectively; 20 μL of MTT solution (thiazolyl blue tetrazolium bromide, Sigma) were added to each well from MTT stock solution (5 mg /mL) after incubation. After 4 h of incubation at 37 °C, 100 μL of sodium dodecyl sulfate (SDS) solution (10% in 0.01 M HCl) was added to each well and all plates were incubated at 37 °C overnight. Finally, cell growth was determined by measuring the optical density (OD) at 540 nm (ref. 630 nm) with Multiscan EX ELISA reader (Thermo Labsystems, Cheshire, WA, USA). 

The inhibition of the cell growth was determined according to the formula mentioned elsewhere [20]. Finally, the cell viability and IC_50_ values for every tested compound and doxorubicin were calculated using GraphPad Prism, version 5.03. 

The selectivity index (SI) was calculated as the quotient between the IC_50_ values in the resistant cells (Colo 320) and the sensitive cells (Colo 205). A compound was considered strongly selective if SI > 6 and moderately selective if 3 < SI < 6 [22].

### 4.5. Antibacterial and Antifungal Activities

The antimicrobial activities of the compounds were determined according to the protocol described in the literature, employing a Universal Microplate Reader, EL800, Biotech Instruments, Inc. Highland Park, Winooski, VT, USA [23,24,25]. 

In short words, compounds were dissolved in DMSO and later diluted in the adequate growth media to be evaluated at the final concentrations of 50, 100, 150, and 200 µM. Nutrient Luria Bertani (LB) broth, bacterial basic media, Sabouraud Dextrose (SD), and Yeast Peptone Dextrose (YPD) were exploited as media for *Escherichia coli*, *Staphylococcus carnosus*, *Candida albicans*, and *Saccharomyces cerevisiae*, respectively. In all cases, the final concentration of DMSO was 1% or less. A mixture of penicillin, streptomycin, and amphotericin B (4 U, 0.4 µg/mL and 10 µg/mL, respectively) was employed as positive control for *S. carnosus* and *E. coli*, while ketoconazole was utilized as the positive control for *C. albicans* and *S. cerevisiae*. The untreated culture in medium was employed as the negative control for each organism; 1% DMSO stock solution was used as a solvent control. All experiments were performed in triplicates and on three independent occasions (*n* = 9) [23,24,25].

### 4.6. Nematicidal Activity

The soil nematodes *S. feltiae* were freshly purchased for experiment from Sautter and Stepper GmbH (Ammerbuch, Germany) and stored at 4 °C in the dark. The assay was performed according to the protocols described in the literature [24]. Results were expressed as mean ± standard deviation (SD) and their statistical significance values were determined by one-way ANOVA followed by Tukey’s multiple comparison test. To consider data statistically significant, the *p* value should be ≤ 0.05. 

### 4.7. MDR Efflux Pump Inhibition

The efflux pump inhibition was determined measuring the rhodamine 123 (R123) fluorescence-based detection assay by flow cytometry. This assay was performed according to protocol previously described in the literature [20,26]. This is a fluorescence-based detection assay which uses verapamil as a reference inhibitor of P-gp efflux pump. The colonic adenocarcinoma cells (in this work the doxorubicin-sensitive Colo 205 and the multidrug resistant Colo 320) were adjusted to a density of 2 × 10^6^/mL. The cells were re-suspended in serum-free RPMI 1640 medium and distributed in 0.5 mL aliquots into Eppendorf centrifuge tubes. Compounds (1 μL and 10 μL from a stock solution of 1 mM) were added to the medium to achieve solutions with final concentrations of 2 μM and 20 μM respectively, followed by incubation of the sample for 10 min at room temperature. R123 (10 μL, 5.2 μM final concentration) was added to the samples and the cells were incubated for 20 min at 37 °C followed by washing twice with phosphate buffered saline (PBS) and re-suspension in 1 mL PBS for analysis. The fluorescence intensity of the cell population was measured with a Partec CyFlow flow cytometer (Partec, Munster, Germany). Verapamil (20 μM) was employed as a positive control. The mean fluorescence intensity (%) for the treated MDR Colo 320 and sensitive Colo 205 cell lines as compared to the untreated cells was calculated. The fluorescence activity ratio (FAR) was calculated employing the following equation [20,26]:

FAR = (MDR_treated_/MDR_control_)/(sensitive_treated_/ sensitive_control_) (1)

FAR Quotient % was calculated as follows:

Quotient = (FAR_compound_/FAR_verapamil_) × 100 (2)

### 4.8. Statistical Analysis

All data presented in this work (the antimicrobial, the antifungal, and the nematicidal activities) were expressed as the standard error of the mean (± SEM). For nematicidal activity, the statistical significance values were determined by one-way ANOVA followed by Tukey’s multiple comparison test. A value of *p* ≤ 0.05 was considered statistically significant. For antimicrobial and antifungal activities, data were analyzed by two-way ANOVA, and Bonferroni post hoc test. Statistical significance values were set at * *p* < 0.05, ** *p* < 0.01, and *** *p* < 0.001. GraphPad Prism (Version 5.03, GraphPad Software, USA) was employed for data analysis and to generate charts.

## 5. Conclusions

The search for new anticancer drugs and adjuvants in cancer therapy is an important challenge in pharmaceutical sciences. In the light of the latest research, selenium derivatives bring hope for new solutions which can help in the fight against cancer. As a part of this study, the mechanism of action of novel flavonoid derivatives (in relation to the P-gp MDR efflux pump) was identified. The inhibitory character of the synthetic flavones and bioisosteric analogues against P-gp was confirmed. Moreover, the results of the rhodamine 123 accumulation assay demonstrated a dose-dependent inhibition of P-gp by the flavonoids and analogues. Three compounds (**4**, **5**, and **6**) demonstrated a beneficial, potent inhibitory activity much stronger than verapamil. The results affirmed that the tested flavone derivatives and bioisosteric analogues can successfully be proposed as P-gp efflux pump inhibitors in the therapy of MDR cancer treatment. Moreover, it has been confirmed that all of the tested compounds may serve as promising adjuvants in the cancer treatment as they possess P-gp efflux pump-modulating activity.

## Figures and Tables

**Table 1:** Cytotoxic activity of flavones, thioflavones, and selenoflavones (and bioisosteric analogues).

Cpd	A—Colo 205			B—Colo 320			SI A/B		C—MRC-5			SI C/A	SI C/B
	IC_50_ (μM)	SD ±		IC_50_(μM)	SD ±				IC_50_ (μM)	SD ±			
**1**	>100	-		>100	-		-		>100	-		-	-
**2**	>100	-		>100	-		-		>100	-		-	-
**3**	>100	-		>100	-		-		>100	-		-	-
**4**	>100	-		>100	-		-		>100	-		-	-
**5**	>100	-		>100	-		-		>100	-		-	-
**6**	>100	-		>100	-		-		>100	-		-	-
**DOXO**	1.56	0.03		6.45	0.19				>10				

SD: standard deviation, SI: selectivity index.

**Table 2:** Antiproliferative activity screening of the flavones and bioisosteric analogues. SD: standard deviation, SI: selectivity index.

Cpd	A—Colo 205			B—Colo 320			SI A/B
	IC_50_ (μM)	SD ±		IC_50_ (μM)	SD ±		
**1**	>100	-		>100	-		-
**2**	>100	-		>100	-		-
**3**	>100	-		>100	-		-
**4**	>100	-		>100	-		-
**5**	>100	-		>100	-		-
**6**	>100	-		>100	-		-
**DOXO**	0.24	0.03		0.14	0.03		

**Table 3:** P-gp efflux pump inhibitory activity of compounds against MDR Colo 320 colon adenocarcinoma cells.

Sample	Concentration (μM)	FAR ^1^	FAR Quotient (%) ^2^		Sample	Concentration (μM)	FAR	FAR Quotient (%) ^3^
**1**	2	1.14	34.92		**1**	20	1.53	46.92
**2**	2	0.97	29.67		**2**	20	1.89	57.83
**3**	2	1.06	32.58		**3**	20	1.45	44.33
**4**	2	1.48	45.42		**4**	20	4.81	**147.50**
**5**	2	1.95	59.92		**5**	20	8.61	**264.17**
**6**	2	1.14	34.92		**6**	20	4.67	**143.33**
**Verapamil**	20	3.26	100.0		**DMSO**	2% V/V	0.80	24.67

^1^ FAR: Fluorescence activity ratio; ^2^ FAR Quotient is the FAR value of the compound divided by the FAR of the verapamil and multiplied by 100. Values above 100% (those more active than verapamil) are highlighted with bold letters. ^3^ Values in bold denote a FAR quotient higher than 100.

## Supplementary Materials

The following are available online at https://www.mdpi.com/1424-8247/13/12/453/s1, Figures S1–S6, containing each one the antimicrobial activities of each one of the compounds **1**–**6**, against Gram-positive bacteria *S. carnosus* (a), Gram-negative bacteria *E. coli* (b), pathogenic yeast *C. albicans* (c) and non-pathogenic yeast *S. cerevisiae* (d).
