# Supplementary material for: Antimicrobial, Anticancer and Multidrug-Resistant Reversing Activity of Novel Oxygen-, Sulfur- and Selenoflavones and Bioisosteric Analogues
Source: Pharmaceuticals (Basel). 2020 Dec 11;13(12):453. doi: 10.3390/ph13120453 (PMC7763008; doi:10.3390/ph13120453)
Supplement: Supplementary file 1 [file pharmaceuticals-13-00453-s001.pdf]

Antimicrobial, anticancer and multidrug-resistant reversing activity of novel oxygen-, sulfur- and selenoflavones and bioisosteric analogues

Małgorzata Anna Marć*, Annamária Kincses, Bálint Rácz, Muhammad Jawad Nasim, Muhammad Sarfraz, Carlos Lázaro-Milla, Enrique Domínguez-Álvarez, Claus Jacob, Gabriella Spengler and Pedro Almendros

Supplementary material: antimicrobial and antifungal activities of the tested oxygen-, sulfur- and selenoflavonones and bioisosteric analogues

Compound 1

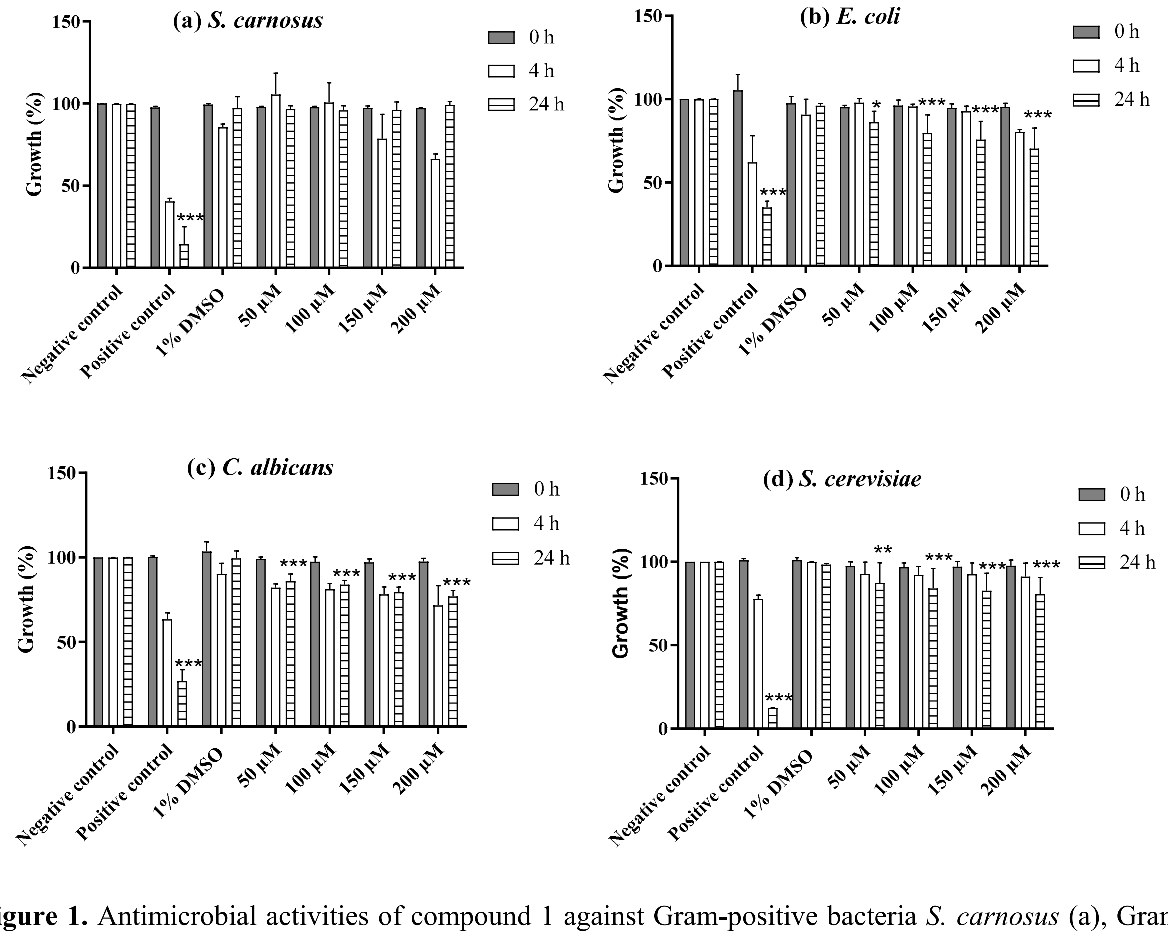

Figure 1. Antimicrobial activities of compound 1 against Gram-positive bacteria *S. carnosus* (a), Gram-negative bacteria *E. coli* (b), pathogenic yeast *C. albicans* (c) and non-pathogenic yeast *S. cerevisiae* (d). A mixture of penicillin, streptomycin, and amphotericin B (4 U, 0.4 µg/mL, and 10 µg/mL, respectively) was employed as positive control for antibacterial assays whilst ketoconazole was exploited as positive control yeast-based assays. Growth cultures of the respective microorganisms with medium were employed as negative control in each experiment.

Compound 2

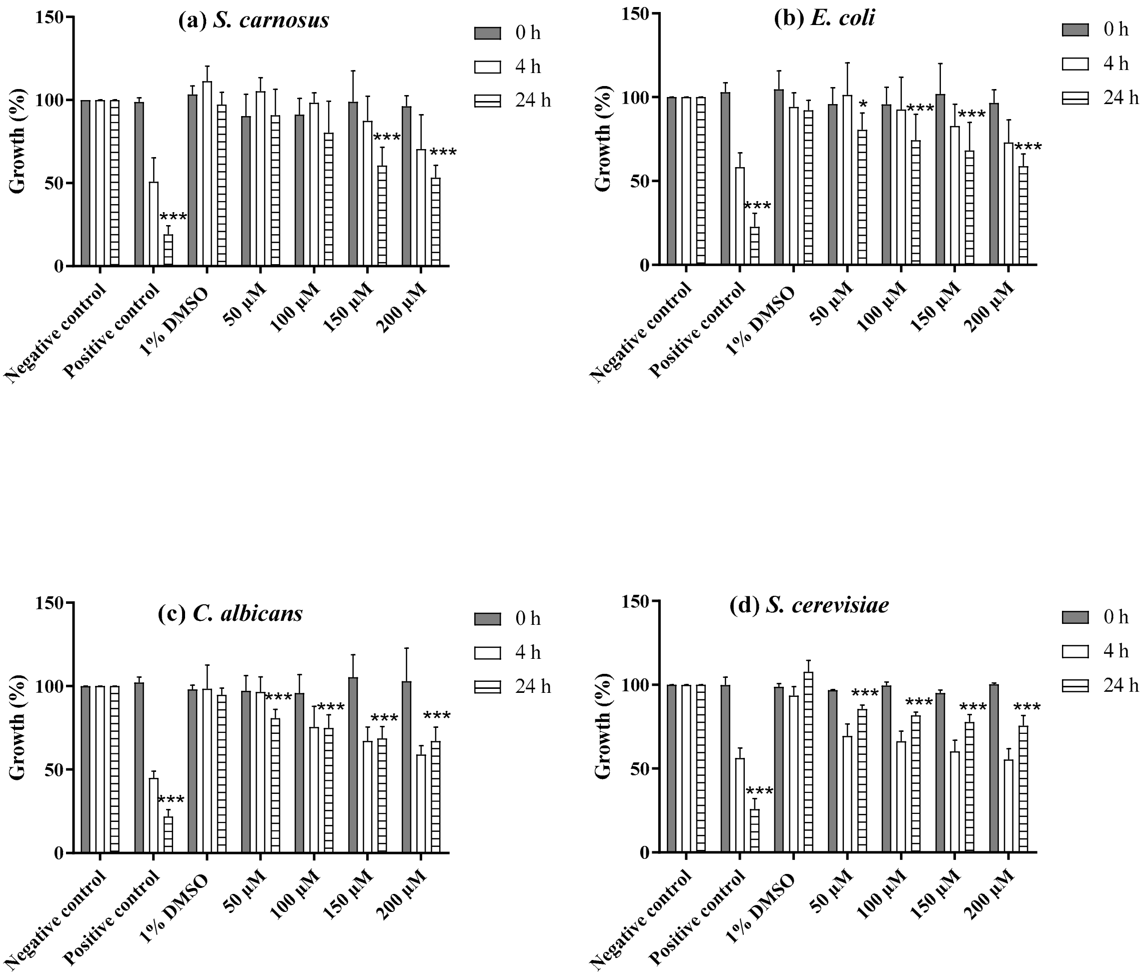

Figure 2. Antimicrobial activities of compound 2 against Gram-positive bacteria *S. carnosus* (a), Gram-negative bacteria *E. coli* (b), pathogenic yeast *C. albicans* (c) and non-pathogenic yeast *S. cerevisiae* (d). A mixture of penicillin, streptomycin, and amphotericin B (4 U, 0.4 µg/mL, and 10 µg/mL, respectively) was employed as positive control for antibacterial assays whilst ketoconazole was exploited as positive control yeast-based assays. Growth cultures of the respective microorganisms with medium were employed as negative control in each experiment.

Compound 3

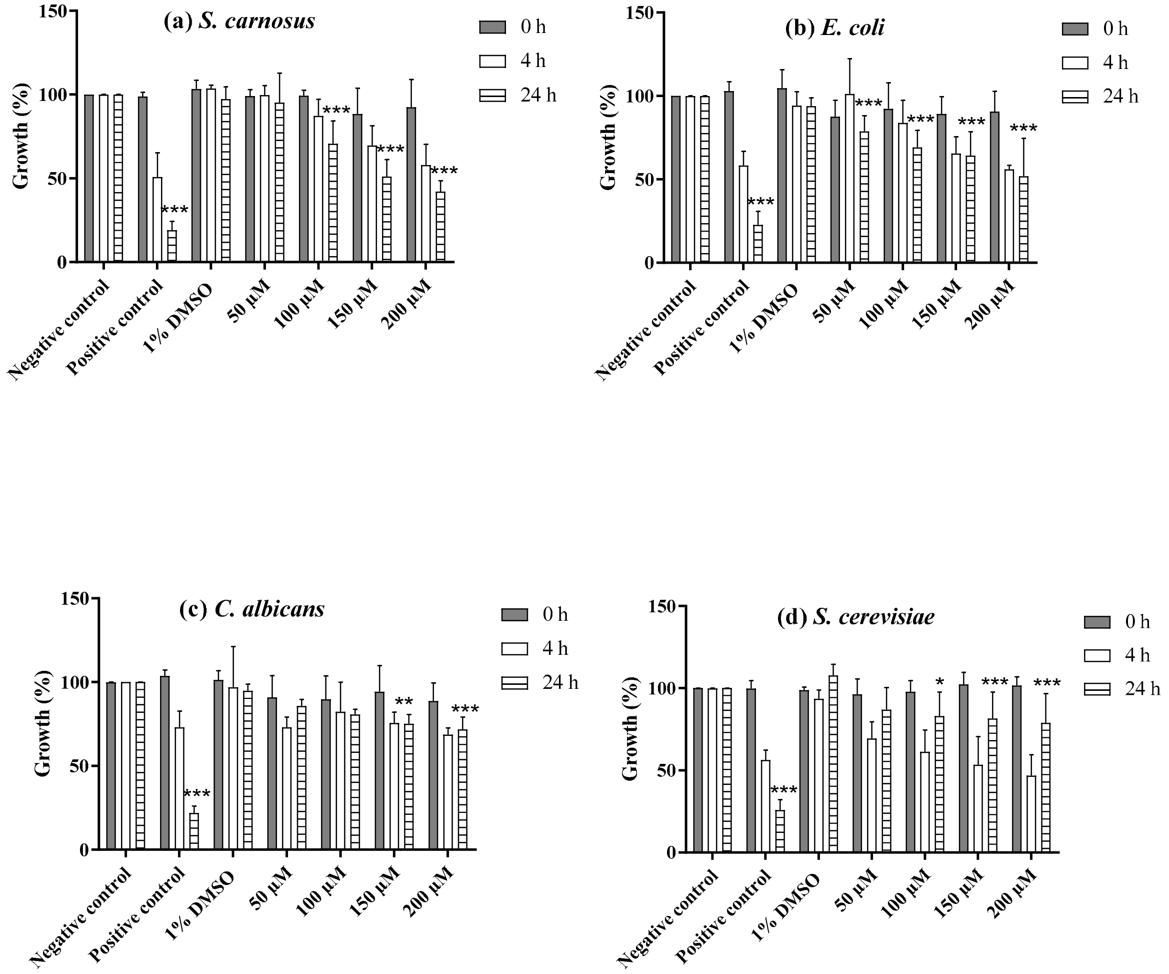

Figure 3. Antimicrobial activities of compound 3 against Gram-positive bacteria *S. carnosus* (a), Gram-negative bacteria *E. coli* (b), pathogenic yeast *C. albicans* (c) and non-pathogenic yeast *S. cerevisiae* (d). A mixture of penicillin, streptomycin, and amphotericin B (4 U, 0.4 µg/mL, and 10 µg/mL, respectively) was employed as positive control for antibacterial assays whilst ketoconazole was exploited as positive control yeast-based assays. Growth cultures of the respective microorganisms with medium were employed as negative control in each experiment.

Compound 4

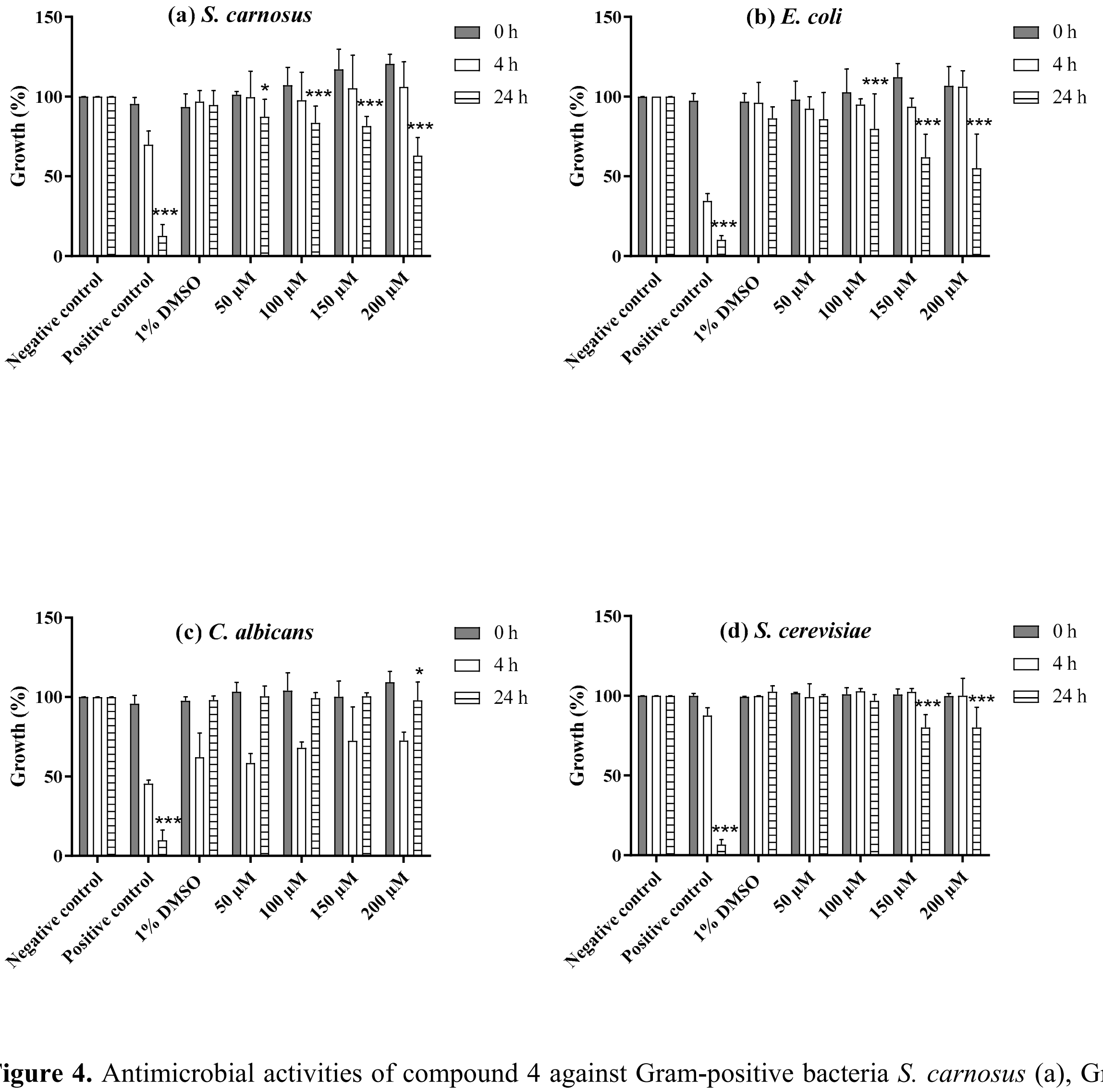

Figure 4. Antimicrobial activities of compound 4 against Gram-positive bacteria *S. carnosus* (a), Gram-negative bacteria *E. coli* (b), pathogenic yeast *C. albicans* (c) and non-pathogenic yeast *S. cerevisiae* (d). A mixture of penicillin, streptomycin, and amphotericin B (4 U, 0.4 µg/mL, and 10 µg/mL, respectively) was employed as positive control for antibacterial assays whilst ketoconazole was exploited as positive control yeast-based assays. Growth cultures of the respective microorganisms with medium were employed as negative control in each experiment.

Compound 5

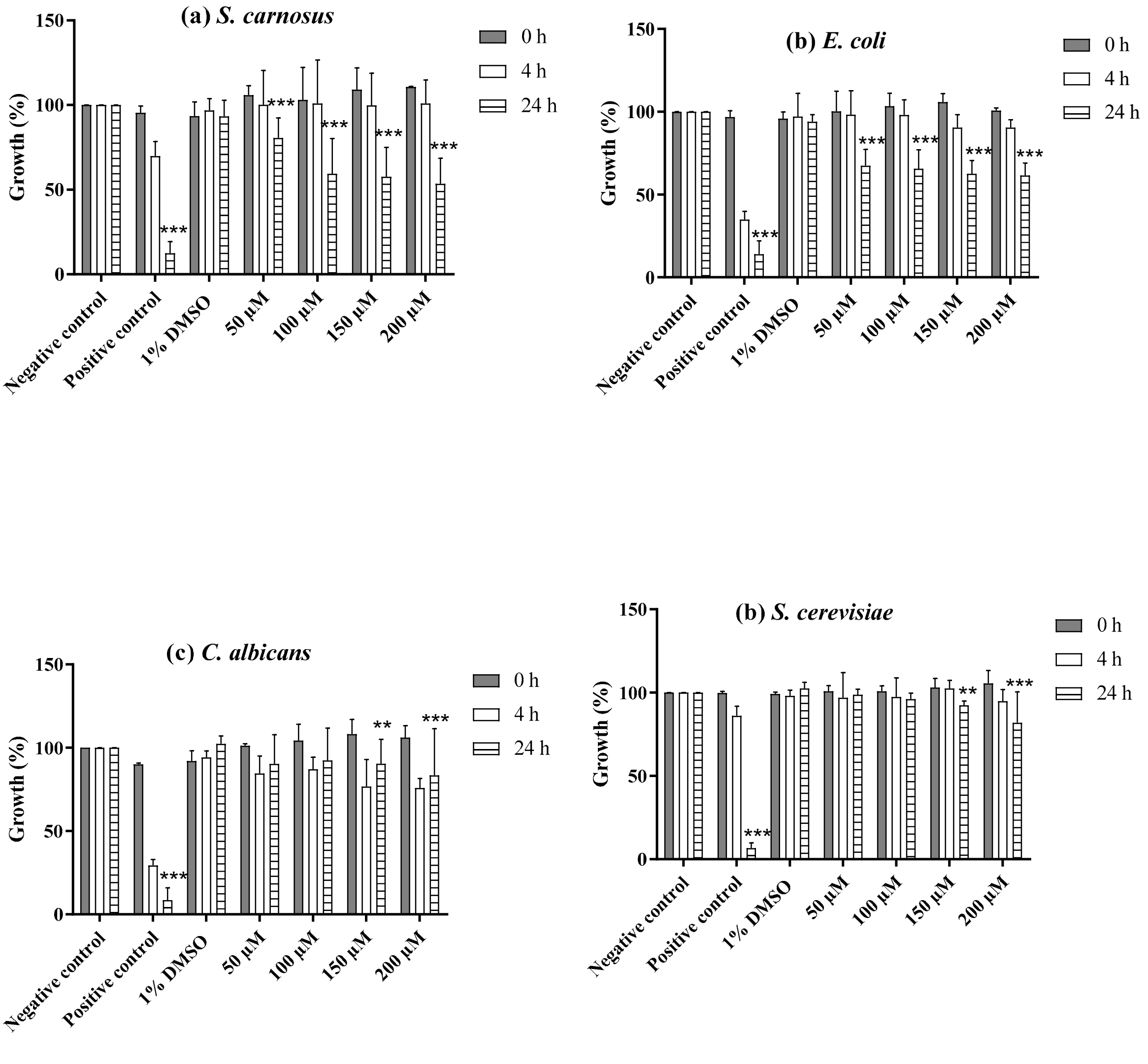

Figure 5. Antimicrobial activities of compound 5 against Gram-positive bacteria *S. carnosus* (a), Gram-negative bacteria *E. coli* (b), pathogenic yeast *C. albicans* (c) and non-pathogenic yeast *S. cerevisiae* (d). A mixture of penicillin, streptomycin, and amphotericin B (4 U, 0.4 µg/mL, and 10 µg/mL, respectively) was employed as positive control for antibacterial assays whilst ketoconazole was exploited as positive control yeast-based assays. Growth cultures of the respective microorganisms with medium were employed as negative control in each experiment.

Compound 6

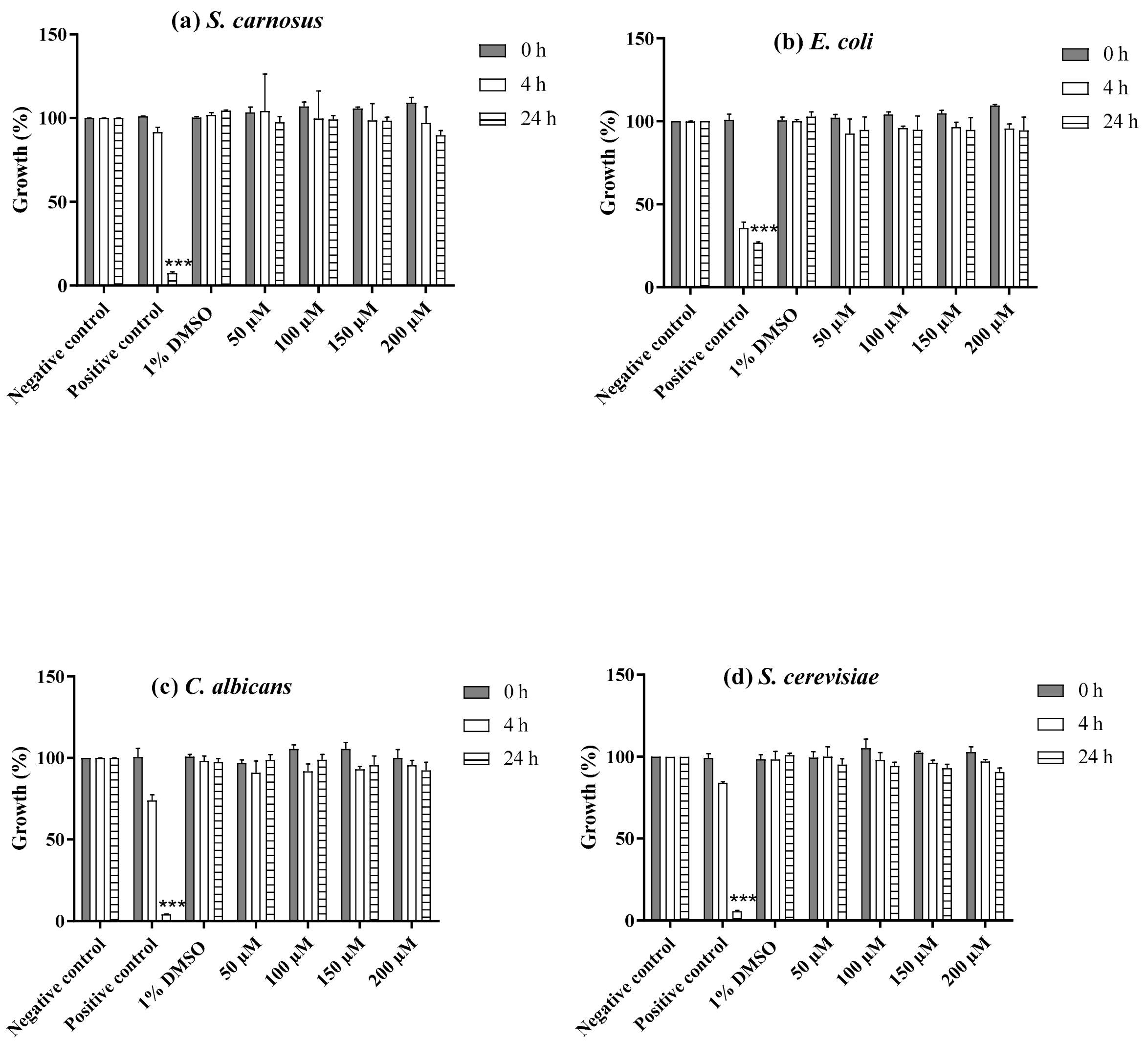

Figure 6. Antimicrobial activities of compound 6 against Gram-positive bacteria *S. carnosus* (a), Gram-negative bacteria *E. coli* (b), pathogenic yeast *C. albicans* (c) and non-pathogenic yeast *S. cerevisiae* (d). A mixture of penicillin, streptomycin, and amphotericin B (4 U, 0.4 µg/mL, and 10 µg/mL, respectively) was employed as positive control for antibacterial assays whilst ketoconazole was exploited as positive control yeast-based assays. Growth cultures of the respective microorganisms with medium were employed as negative control in each experiment.
